# Grating Bio-Microelectromechanical Platform Architecture for Multiple Biomarker Detection

**DOI:** 10.3390/bios14080385

**Published:** 2024-08-09

**Authors:** Fahimeh Marvi, Kian Jafari, Mohamad Sawan

**Affiliations:** 1 CenBRAIN Neurotech Center of Excellence, School of Engineering, Westlake University, Hangzhou 310030, China; fahime.marvi@westlake.edu.cn; 2 Institute of Advanced Technology, Westlake Institute for Advanced Study, Hangzhou 310024, China; 3 Mechanical Engineering Department, Faculty of Engineering, Université de Sherbrooke (UdeS), 2500 Boul. de l’Université, Sherbrooke, QC J1L 2G7, Canada; kian.jafari@usherbrooke.ca; 4 Interdisciplinary Institute for Technological Innovation (3IT), Université de Sherbrooke (UdeS), Sherbrooke, QC J1K 2R1, Canada

**Keywords:** BioMEMS sensing, metamaterial structure, optical measurements, biological events, biomarkers

## Abstract

A label-free biosensor based on a tunable MEMS metamaterial structure is proposed in this paper. The adopted structure is a one-dimensional array of metamaterial gratings with movable and fixed fingers. The moving unit of the optical detection system is a component of the MEMS structure, driven by the surface stress effect. Thus, these suspended optical nanoribbons can be moved and change the grating pattern by the biological bonds that happened on the modified cantilever surface. Such structural variations lead to significant changes in the optical response of the metamaterial system under illuminating angled light and subsequently shift its resonance wavelength spectrum. As a result, the proposed biosensor shows appropriate analytical characteristics, including the mechanical sensitivity of S_m_ = 11.55 μm/Nm^−1^, the optical sensitivity of S_o_ = Δλ/Δd = 0.7 translated to S_o_ = Δλ/Δσ = 8.08 μm/Nm^−1^, and the quality factor of Q = 102.7. Also, considering the importance of multi-biomarker detection, a specific design of the proposed topology has been introduced as an array for identifying different biomolecules. Based on the conducted modeling and analyses, the presented device poses the capability of detecting multiple biomarkers of disease at very low concentrations with proper precision in fluidic environments, offering a suitable bio-platform for lab-on-chip structures.

## 1. Introduction

Early diagnosis of chronic diseases, such as neuronal disorders and any types of cancers, is a significant concern in global health to harness their severe impacts on communities. These diseases can usually be diagnosed by monitoring several biomarkers, which require very sensitive instruments for early, accurate detection. As a consequence, finding affordable and non-invasive techniques to diagnose such diseases has gained much attention recently [1,2]. According to recent advances, label-free biosensors can be promising alternatives for efficient and cost-effective diagnosis of various diseases [3,4,5].

Nonetheless, these devices, which can measure the concentration of biomarkers based on the physiochemical interactions and their biological properties, inevitably need enhancement in their performance to increase their reliability and reproducibility so that they can be commercialized for clinical practices [6,7,8].

While electrochemical biosensors are popular structures in the context of disease detection, particularly in wearable devices, several limitations, such as the need for sample preparation in most cases, high sensitivity to unwanted changes in the test environment, and the use of nanostructures or nanoparticles to improve their performance, have challenged their reliability and reproducibility [9,10,11]. In optical biosensors, despite the advantages of emerging optical technologies, the relatively low detection limit in photonic structures and low resolution, along with the high implementation complexities in plasmonic systems, have limited their usage to become routine devices in clinical diagnostics [12,13]. Recently, colorimetric biosensors, as affordable and sensitive optical mechanisms, are gaining more attention in detecting various analytes [14]. However, they also need to enhance their performance to overcome some challenges, including low stability, high sensitivity to environmental changes (i.e., pH), and low reproducibility [15,16].

On the other hand, BioMEMS devices are also recognized as highly sensitive structures in biosensing applications. These electromechanical sensors can detect biological species by measuring the dynamic or static deflection of their transducers [17]. MEMS resonating biosensors (dynamic structures), which are based on the mass changes in the transducer, suffer from a substantial decline in quality factor due to the damping effect in the fluids. In these structures, the correlation of binding events on the surface of the oscillating MEMS cannot affect the sensor performance, resulting in a low complexity of immobilizing biological receptors [18,19]. However, these topologies require surface modification or preparation of the target sample to reduce the non-specific interactions caused by the adsorption of interfering molecules on the surface [20]. In static MEMS biosensors, due to the underlying mechanism of surface stress generation, the surface functionalization step is of great importance in sensor operation. Connection and continuity of biological interactions on the MEMS surface are necessary for such structures so that they can release the desired surface energy and deflect the movable part [21,22,23].

This static displacement can be subsequently measured by electrical or optical signals. Although surface stress-based biosensors can be developed as integrated systems using electrical measurements, such as capacitive or piezoresistive methods, low sensitivity and high noise make challenges for the accurate detection of biomarkers in these devices [24,25]. In optical detections, despite the high sensitivity, the need for a reference cantilever, as well as the complexity of implementing a free-space detection setup, are some of the significant challenges for this type of biosensor [26,27].

In this paper, the design of a MEMS biosensing platform based on a one-dimensional grating metamaterial is proposed to address the concerns raised. In the present structure, a highly sensitive cantilever used as a BioMEMS transducer causes structural changes in the proposed metamaterial design, which leads to strong changes in the wavelength spectrum of this periodic optical pattern. Therefore, very small changes in the concentration of biomarkers produce nanomechanical changes in the suspended structure and, thus, provide a desired wavelength shift in the optical output. Also, given the importance of multi-biomarker identification in the diagnosis of chronic diseases, including neurological disorders, an array design of the proposed biosensor for multiplexed biomarker analysis without increasing the complexity of the optical detection system is presented. Therefore, the proposed platform can be a valuable device in the diagnosis of chronic diseases as well as drug monitoring applications to evaluate their efficacy. The following sections are dedicated to providing detailed descriptions of the sensor performance, a comprehensive analysis of its MEMS and optical characteristics, and a comparison with recent contributions in the related fields.

## 2. Operating Principles of the Proposed Optical MEMS Biosensor

**Figure 1:**
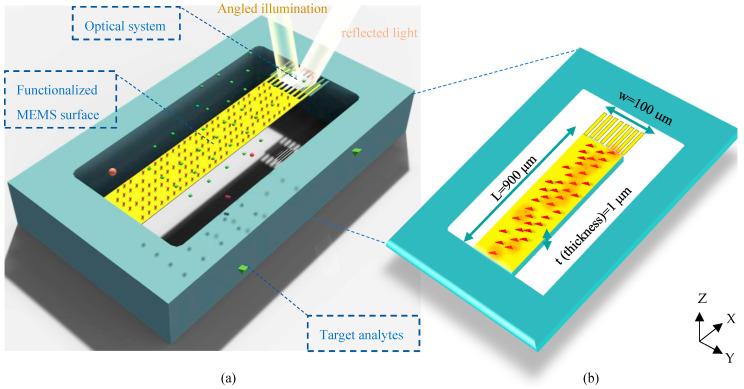
Schematic of the proposed optical BioMEMS platform for biosensing of target analytes: (**a**) 3D design of the biosensor topology, and (**b**) a description of the physical parameters of its suspended MEMS framework (under displacements in the Z-direction).

The architecture of the proposed structure can be seen in Figure 1a. In this surface stress-based opto-mechanical sensor, a laser source in a desired spectrum should be appropriately set up to illuminate the optical metamaterial structure, resulting in a resonance wavelength of metamaterial cells. The MEMS topology enables structural changes in the optical metamaterial grating system to yield a significant shift in the resonant wavelength due to small changes in the concentration of biospecies. This shift is detected in the sensor output by an appropriate photodetector. In this section, the mechanism of the structure is described.

### 2.1. MEMS Analysis

To obtain high sensitivity in biosensing, the proposed sensor is designed based on the surface stress generation mechanism to quantify biomolecules. In the MEMS skeleton of this structure, a highly sensitive silicon cantilever supporting movable nanoribbons of the optical grating is coated with a gold layer to serve as an opto-biomechanical transducer.

In this transducer, one side of the cantilever (usually the top side) should be activated by immobilizing bioreceptors, and the other side is passivated. Thus, the thiolated Self-Assembled Monolayer (SAM) procedure can be properly utilized to functionalize the top side of the sensor surface by biorecognition elements using thiol–gold interactions. Please note that the active site involved in this process is the cantilever part of the proposed design. It means that considering the nanoscale size of grating nanoribbons and the correlation basis of surface stress generation, biological interactions on gold nanoribbons can be properly neglected. The binding events on the surface of the MEMS cantilever form a continuous network of active sites (Figure 1b), inducing energy to the activated surface. The continuity of these energies produces a force on one side that statically moves the cantilever based on its stiffness. Considering the spring constant of the designed transducer (*k*), the mechanical sensitivity of the sensor is calculated by Stoney’s equation as follows [20]:

(1) $$k=\frac{Ewt^{3}}{4L^{3}}=0.0054\frac{N}{m}$$

(2) $$S_{m}=\frac{∆Z}{∆\sigma }=\frac{3L^{2}(1-\upsilon )}{Et^{2}}=11.8\frac{\mu m}{Nm^{-1}}$$

**Figure 2:**
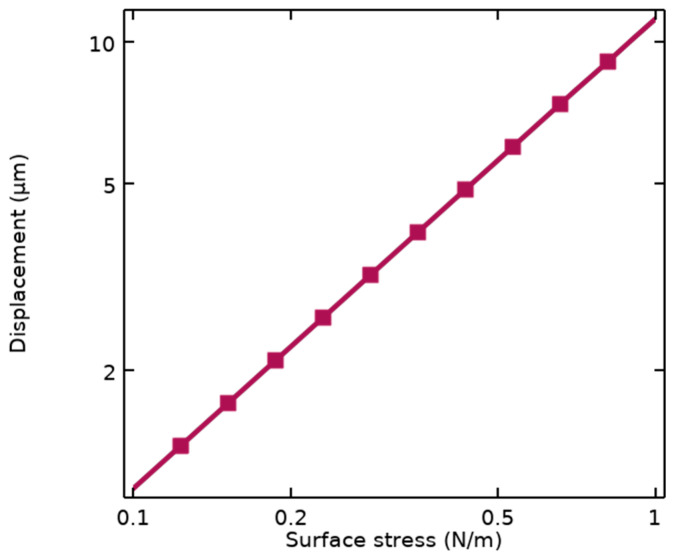
Simulated mechanical sensitivity of the suspended cantilever under applied surface stresses.

Stoney’s equation depends on the dimensional parameters of the cantilever illustrated in Figure 1b, including *L* and *t*, as well as properties of the structural materials such as Young’s modulus (*E*) and Poisson’s ratio (*ν*). This sensitivity is also simulated using FEM analysis by COMSOL Multiphysics, resulting in S_m_ = 11.55 μm/Nm^−1^ represented in Figure 2.

Therefore, according to this sensitivity, the grating nanoribbons connected to the end of the MEMS transducer undergo maximum displacement, leading to changes in the optical pattern.

**Figure 3:**
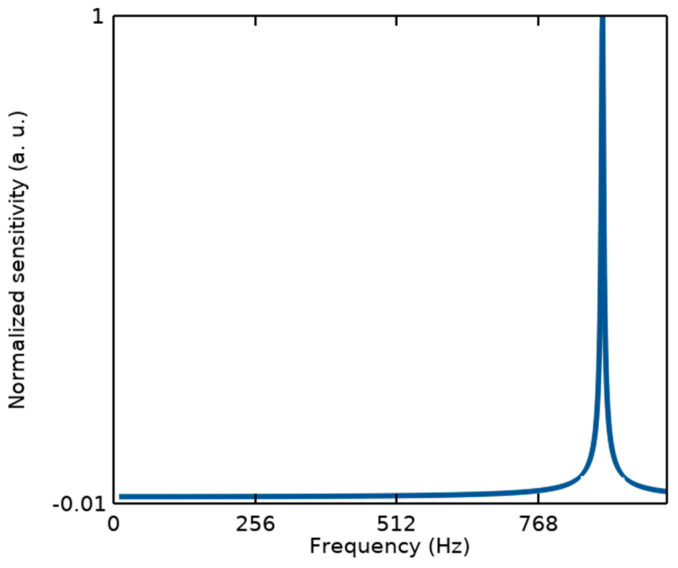
Modal analysis of the proposed BioMEMS structure to estimate its bandwidth considering the first resonant frequency of f_r1_ = 876.9 Hz.

It should be noted that from the mechanical point of view, since the measurements of the proposed structure are performed in the static mode, the sensor linearity can be defined within its bandwidth (BW) related to the first resonance frequency, as illustrated in Figure 3. While other resonance modes with a large difference from the first one cannot affect its performance.

(3) $$f_{r}=\frac{1}{2\pi }\sqrt{\frac{k}{m}},\;BW\approx \frac{f_{r}}{5}\approx 180\;\mathrm{Hz}$$

### 2.2. Mechanism of the Optical System

**Figure 4:**
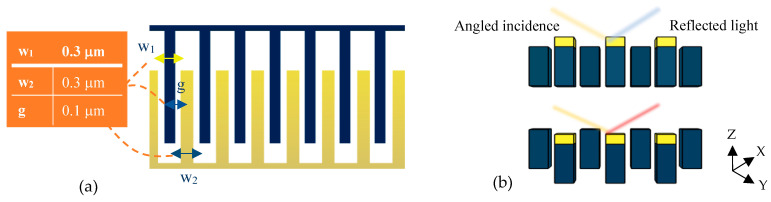
Optical pattern of the proposed grating metamaterials: (**a**) a top view for physical parameter description and (**b**) a cross-section view to explain the tuning mechanism under cantilever displacement.

The tunable optical system of the present design is depicted in Figure 4a. This optical setup is a metamaterial one-dimensional grating consisting of suspended and fixed nanoribbons. The fixed parts are bare silicon fingers, while the suspended nanoribbons are coated with a thin layer of gold and separated from the fixed ones by a small gap of 100 nm.

According to the strong optical confinement of photonic grating patterns and, on the other hand, the high sensitivity of plasmonic structures, the purpose of proposing this metamaterial grating is to simultaneously achieve a high sensitivity and an appropriate quality factor for the optical measurements. Therefore, the proposed design has been optimized by several parametric analyses in the Wave Optics module of COMSOL Multiphysics version 6.1. In this optimization, diverse parameters, such as the incident angle of light on the metamaterial surface and the width of the grating nanoribbons in a constant period, play important roles in obtaining appropriate characteristics.

**Figure 5:**
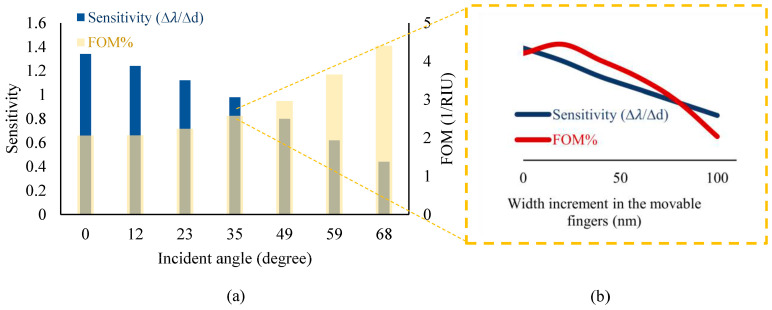
A compromise between the sensitivity and Figure of Merit (FOM) of the sensor (based on minimizing the FWHM) by optimizing several effective parameters of the proposed optical design, including (**a**) the incident angle and (**b**) the width of movable nanoribbons under different incident angles.

**Figure 6:**
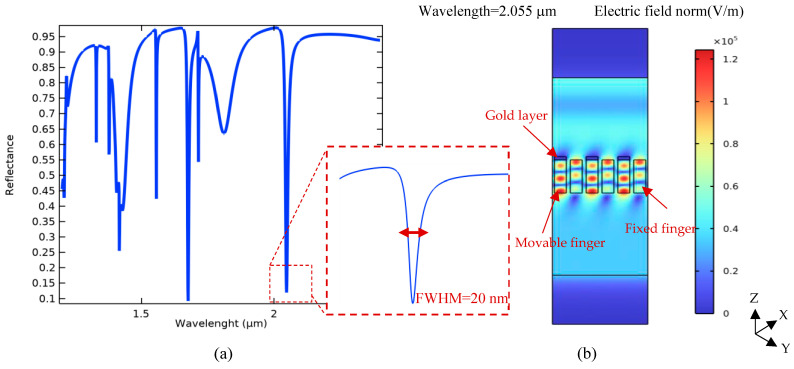
Simulated optical spectrum of the proposed tunable metamaterial gratings: (**a**) different resonances of the optical pattern in its wavelength spectrum under TE mode excitation (incident angle of 50 degrees), and (**b**) a cross-view of the electric field distribution of incident light in the desired resonant wavelength for a part (three cells) of the periodic pattern (λ = 2.055 μm).

Therefore, according to the obtained results plotted in Figure 5, the proposed optical pattern has been finally modeled under illuminating TE mode waves with a radiation angle of 50° and the same width of grating nanoribbons at an 800 nm period. In the neutral state, the position of Au and Si nanoribbons is the same, yielding multiple resonances in the wavelength spectrum of the proposed system, as shown in Figure 6. In this pattern, the resonance occurred at the wavelength of 2.055 μm, which is targeted as the reference sensing wavelength. This is due to the fact that the biosensor shows higher sensitivity in this wavelength compared to others, and also, there is no resonance in a spectrum surrounding this particular wavelength. It can facilitate wavelength modulation by an array of the proposed platform for the simultaneous detection of multiple biomarkers, which is explained in the next section.

In the optical mechanism of the sensor, the equipped BioMEMS transducer serves to tune the grating pattern. Accordingly, by deflecting the sensitive cantilever due to any changes in the concentration of the target analytes, the free nanoribbons connected to its end are placed in a different position from the silicon nanoribbons and cause significant structural changes (Figure 4b) in the proposed metamaterial grating.

**Figure 7:**
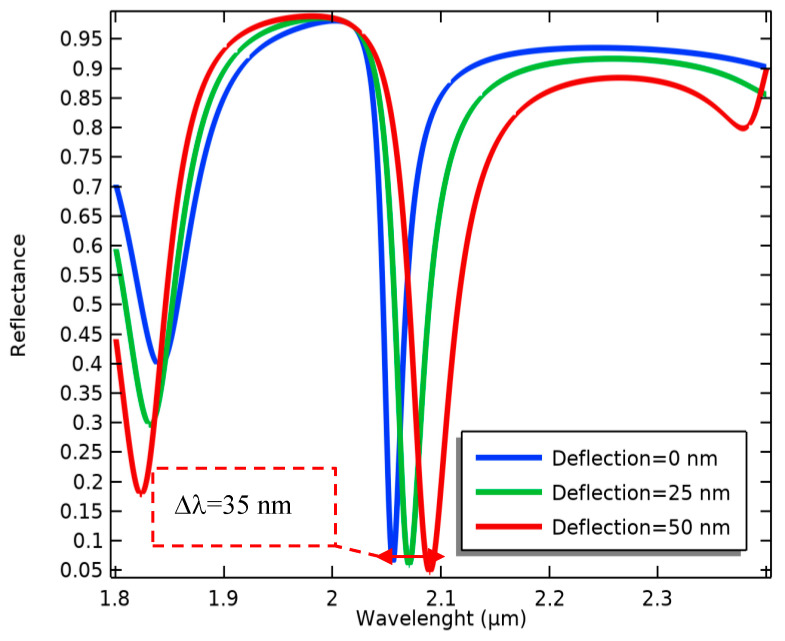
Wavelength changes in the movable grating metamaterials induced by several cantilever deflections.

Consequently, the optical resonance frequency is changed (Figure 7), evaluating the biological alterations in the proposed biosensor. According to this process, the wavelength sensitivity of the present optical system in terms of the changes in the MEMS transducer is calculated as follows:

(4) $$S_{\lambda }=\frac{\Delta \lambda }{∆d}=0.7$$

Considering the sensitivity of the MEMS transducer (the simulation result), the optical sensitivity in the sense of surface stress changes is finally obtained as follows:

(5) $$S_{O}=\frac{\Delta \lambda }{∆\sigma }=\frac{∆\lambda }{∆d}\times \frac{∆d}{∆\sigma }=8.08\;\frac{\mu m}{Nm^{-1}}$$

In addition, the quality factor of the proposed optical structure can be determined by the Full Width at Half Maximum (FWHM) factor as follows:

(6) $$Q=\frac{\lambda_{r}}{FWHM}=102.7.$$

Based on the reported data, the presented optical structure, which determines the detection sensitivity of the surface stress-based biosensor, is highly sensitive to concentration changes on the surface of the cantilever (besides its appropriate quality factor). This highlights the ability of the proposed device to detect very low concentrations of biomarkers.

### 2.3. Multiple Biomarker Detection

Given the significance of detecting multiple biomarkers in label-free biosensors for diagnosis of chronic diseases, such as Alzheimer’s disorder or various types of cancers, an array design of the present structure is proposed to identify different biomarkers.

**Figure 8:**
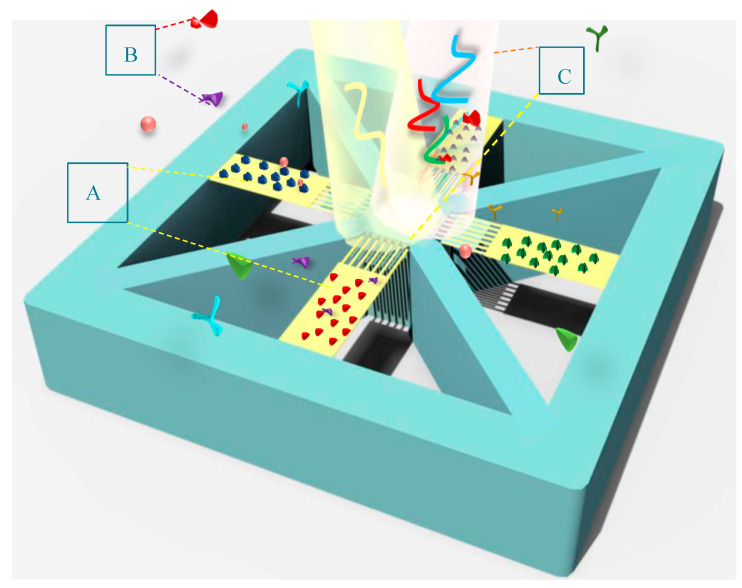
A schematic of the proposed BioMEMS platform for multiplex detection of biomarkers, highlighting: (**A**) an array of functionalized cantilevers by different specific bioreceptors; (**B**) biological markers; and (**C**) illuminated light and its reflection for wavelength modulation.

As indicated in Figure 8, BioMEMS cantilevers (device cells) of the same size make an array structure while supporting grating patterns with different periods. In this design, the fixed nanoribbons are anchored to the four sides of the central body to provide the ability of simultaneous detection of the optical output for four bio-transducers by one detector. By irradiating input light on the surface of the metamaterial gratings with different periods, the reference wavelengths related to each design can be detected in the output spectrum.

**Figure 9:**
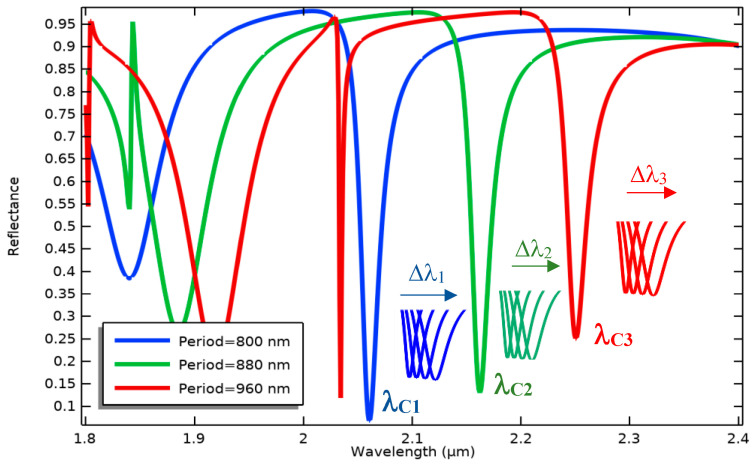
Wavelength modulation of three optical patterns with different grating periods in a specific spectrum to simultaneously measure various concentrations of different biomarkers.

Each cantilever cell functionalized by specific bioreceptors can sensitively transfer concentration changes in a target biomarker to the optical design, which leads to the resonance wavelength shift of the related cell, as seen in Figure 9. These shifts can be differentiated from the changes caused by other biomarkers due to the different reference wavelengths. Therefore, implementing this BioMEMS structure using mass production poses a high potential in biosensing applications to be a cost-effective and efficient device for the diagnosis of a wide range of diseases. It is noteworthy that the proposed biosensor not only has the potential of simultaneous detection of several biomarkers with one detector, considering the wavelength modulation, but also, according to the mechanical design and its framework, immobilizing different biological receptors on the surface of each sensor cell can be easily realized by preparing a multi-chamber microfluidic platform and using the SAM method.

## 3. Comparative Study

Since the proposed structure relies on MEMS grating metamaterial, its performance can be analyzed in the field of optical MEMS. As the results show, the proposed optical BioMEMS sensor has advantages over the previous studies in this context, which are summarized in Table 1 and Table 2. (simulation-based optical and optical MEMS designs).

According to Table 1, the BioMEMS transducer of the proposed system, as a cantilever supporting grating nanoribbons, has a very high mechanical sensitivity, which makes it sensitive to very small changes in biomolecules. On the other hand, by taking advantage of both photonic and optical plasmonic technologies, the proposed optical system shows a very high optical sensitivity and an appropriate quality factor. Notably, obtaining an appropriate quality factor in plasmonic or metamaterial structures is an important issue due to the lossy nature of gold as the main structural material of such systems, addressed in Table 2. Thus, biological interactions can be appropriately converted into optical signals using our topology to quantify the biomolecule concentrations.

In addition, the proposed biosensor can be introduced by an innovative design to operate as an array in the identification of various biomarkers. Such a device not only offers the surface functionalization of each transducer (as a cell of the array) by the simple SAM method but also can realize the measurement of the output signals with one detector.

As a result, considering the mass production of the proposed biosensor based on several micromachinery methods, including lithography, etching, and deposition on SOI wafers, this structure can be remarkable in the early diagnosis of multi-biomarker diseases, particularly chronic diseases such as neurodegenerative disorders or cancers.

## 4. Conclusions

Diagnosing chronic diseases in the early stages or monitoring their progression using cost-effective and efficient methods is of particular importance. For this purpose, in this paper, we aimed to propose an effective structure for advancing label-free biosensors to meet such demands.

The proposed design, which is based on tuning a metamaterial 1D grating by surface stress phenomenon, can detect biological changes with high sensitivity and appropriate precision. In other words, by changing the concentration of the desired analytes, the biological interactions result in a static mechanical signal of the sensitive MEMS part applied to an engineered optical system. Consequently, the proposed sensor shows an ultra-high sensitivity of 8.08 μm wavelength shift per surface stress unit. Also, the quality factor of this optical metamaterial sensor was analytically calculated as Q = 102.7, which is appropriate compared to plasmonic and metamaterial structures.

Furthermore, the mass production of the proposed system using bulk micromachining technology and the possibility of expanding its design as an array make it appealing for molecular diagnostic applications. As a result, an array of the proposed structure has been introduced, relying on wavelength modulation in the resonance spectrum of the optical structure. In this design, taking advantage of the specific topology of the presented sensor and the possibility of surface functionalization of the array using the SAM method, an optical detector can realize simultaneous detection of multiple biomarkers. Accordingly, the proposed platform can be a cost-effective, efficient device for diagnosing chronic diseases as well as therapeutic drug monitoring.

## Figures and Tables

**Table 1:** Performance of the proposed BioMEMS sensor compared to recent contributions in the related field.

Q-Factor	Sensor Sensitivity (μm/Nm^−1^)	Optical Sensitivity	Mechanical Sensitivity (μm/Nm^−1^)	Biosensor
3078	0.2	0.2	1.05	[28]
80	0.6	0.5	1.2	[29]
102.7	8.08	0.7	11.55	This work

**Table 2:** Comparison of the optical mechanism of the proposed biosensor considering different optical methods.

Q-Factor	FWHM (nm)	Optical Method	Biosensor
27	52	Metamaterials	[30]
<60	>12	Plasmonics	[31]
81.58	19.6	2D Photonic crystal	[32]
102.7	20	MEMS grating metamaterials	This work

## Data Availability

Data are contained within the article.
